# The complete mitochondrial genome of spiny spooner *Etisus dentatus* (Herbst, 1785) using high-throughput sequencing

**DOI:** 10.1080/23802359.2021.1891982

**Published:** 2021-03-18

**Authors:** Hongtao Liu, MingHui Shen

**Affiliations:** a Ministry of Education, Key Laboratory of Utilization and Conservation for Tropical Marine Bioresources (Hainan Tropical Ocean University), Sanya, China; b Hainan Provincial Key Laboratory of Tropical Maricultural Technologies, Hainan Academy of Ocean and Fisheries Sciences, Haikou, China

**Keywords:** Etisus dentatus, mitochondrial genome phylogenetic analysis

## Abstract

The whole mitochondrial genome of the spiny spooner *Etisus dentatus* collected from the South China Sea was determined for the first time using high-throughput sequencing. The circular mitogenome of *E. dentatus* is 15,884 bp, with 13 protein-coding genes (PCGs), 22 tRNA genes, two rRNA genes. The base composition is significantly biased (A, G, T, and C was 37.9%, 17.8%, 34.0%, and 10.3%, respectively) with A + T contents of 71.9%. Among 13 PCGs, all PCGs use a normal ATN as the start codon, and ten of them end with TAA or TAG except COX1, COX2 and CYTB gene using a single T as the stop codon. The phylogenetic tree showed that *E. dentatus* forms a cluster *E. anaglyptus* while *Atergatis integerrimus* form a sister group to this cluster.

*Etisus dentatus,* commonly known as spiny spooner, belongs to the family Xanthidae which is one of the most dominant crab families in the Brachyuran with 572 species in 133 genera (De Grave et al. 2009). It is widely distributed in the tropical and subtropical parts of the Indian and Pacific Oceans, and inhabits from the intertidal zone to a depth of 20 m, in reefs or rocky substrates. Although it has been reported to be toxic, but now be eaten in some places like Hainan Island and Paracel Island in China, and its toxicity needs to be further confirmed (Garth and Alcala 1977). Till now mitochondrial genome records of Xanthidae species are inadequate. In the genus *Etisus* of Xanthidae only *E. anaglyptus* was reported (Karagozlu et al. 2018). In the present study, we provide the complete mitochondrial genome analysis of the spiny spooner which will help to clarify the evolution and classification of *Etisus* but also Xanthidae in the Xanthoidea.

The samples of the spiny spooner were collected from Xisha islands, Sansha, China (N16°50'24.37″, E112°21'17.70″), and stored in the marine crustacean specimen room (Hongtao Liu, xmulht@gmail.com) under the voucher number C20200714ED in Qionghai research base of Hainan Academy of Ocean and Fisheries Sciences. The library with an average length of 350 bp was constructed using the NexteraXT DNA Library Preparation Kit, and sequencing was performed on the Illumina Novaseq platform (Total Genomics Solution Limited, SZHT) the 150 bp average length of the generated reads. The whole mitochondrial genome assembled 3.79 G clean reads using the de novo assembler SPAdes 3.11.0 (Bankevich et al. 2012), and annotated using the MITOS (http://mitos.bioinf.uni-leipzig.de/index.py). A phylogenetic analysis was carried out based on the 13 PCGs encoded by 35 Heterotremata mitogenomes available in GenBank using IQ-TREE v1.6.12 (Nguyen et al. 2015) by maximum likelihood (ML) method with 1000 bootstrap replicates, the model is mtMet + F+R5 chosen according to Bayesian information criterion (BIC).

The complete mitogenome of the spiny spooner submitted to the GenBank database (Accession no: MW122295) is 15,884 bp in length. The base composition is 37.9% A, 17.8% G, 34.0% T, and 10.3% C. The 71.9% of (A + T) show a higher preference than G + C. It consists of 13 protein-coding genes (PCGs), 22 tRNA, two rRNA. Four PCGs (ND1, ND4, ND4L and ND5), eight tRNA and two rRNA genes were located on the light strand, the others were encoded by the heavy strand.

The 22 tRNA genes in the mitogenome of the spiny spooner vary from 54 bp to 70 bp. Two tRNA are present more than once: tRNA-Leu and tRNA-Ser both have two type copies respectively. The 12S rRNA is 825 bp, located between tRNA-Val and 16S rRNA; the 16S rRNA is 1361 bp, located between 12S rRNA and tRNA-Leu. There are 11 overlapping regions of 1–7 bp in length. The longest overlapping region is located between ND4 and ND4L. The mitochondrial genome has 14 intergenic sequences varying from 1 to 740 bp in length (Supply Table S1). The largest intergenic sequence is located between 16S rRNA and tRNA-Val. 13PCGs use a normal ATN as the initiation codon, and ten of them end with TAA or TAG except COX1, COX2 and CYTB gene using a single T as the stop codon.

The phylogenetic tree (Figure 1) showed that *E. dentatus* forms a cluster *E. anaglyptus* while *Atergatis integerrimus* form a sister group to this cluster. The results are largely similar to previous studies based on multiple nuclear genes and mitochondrial genes (Lai et al. 2011; Tsang et al. 2014). Taken together, the newly sequenced mitochondrial genome of *E. dentatus* characterized here should contribute to a better understanding of phylogenetic relationships of Xanthoidea species in the Heterotremata, and molecular identification, population genetic and evolutionary biological studies of the spiny spooner.

**Figure 1.**
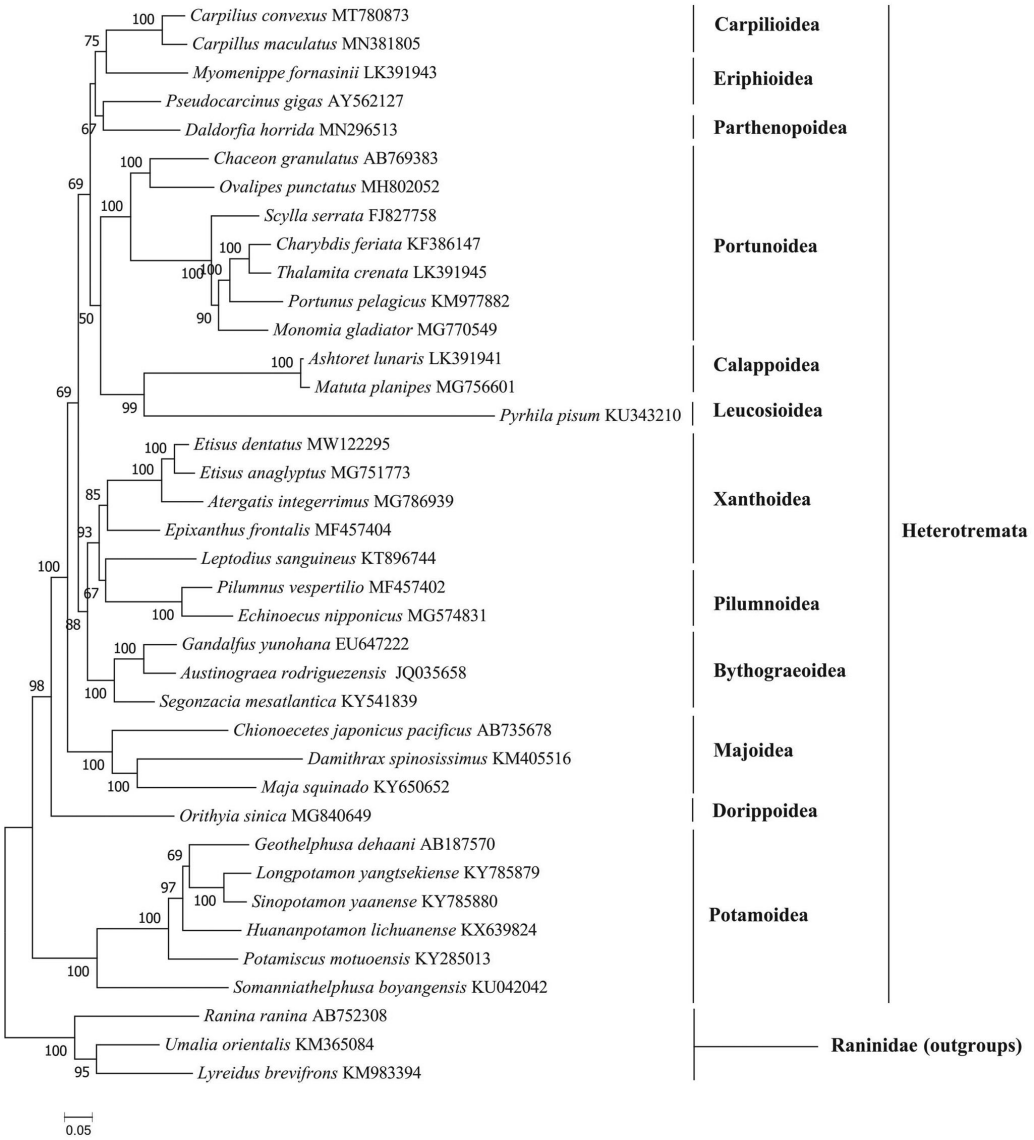
Phylogenetic tree of the complete mitogenome of 35 species in Heterotremata.

The GenBank accession number for each species is indicated after the scientific name. *Lyreidus brevifrons*, *Umalia orientalis* and *Ranina ranina* were used as outgroups.

## Data Availability

The genome sequence data that support the findings of this study are openly available in GenBank of NCBI at (https://www.ncbi.nlm.nih.gov/) under the accession no. MW122295. The associated BioProject, SRA, and Bio-Sample numbers are PRJNA679289, SRR13081715, and SAMN16828461 respectively.
